# Results of a randomized trial of treatment modalities in patients with low or early-intermediate risk prostate cancer (PREFERE trial)

**DOI:** 10.1007/s00432-020-03327-2

**Published:** 2020-09-04

**Authors:** Thomas Wiegel, Peter Albers, Detlef Bartkowiak, Roswitha Bussar-Maatz, Martin Härter, Glen Kristiansen, Peter Martus, Stefan Wellek, Heinz Schmidberger, Klaus Grozinger, Peter Renner, Fried Schneider, Martin Burmester, Michael Stöckle

**Affiliations:** 1 Department of Radiotherapy and Radiation Oncology, University Hospital Ulm, Albert-Einstein-Allee 23, 89081 Ulm, Germany; 2 Department of Urology, University Hospital Düsseldorf, Düsseldorf, Germany; 3 PREFERE Project Management, German Cancer Society, Berlin, Germany; 4 Department of Medical Psychology, University Medical Center Hamburg-Eppendorf, Hamburg, Germany; 5 Institute of Pathology, University Hospital Bonn, Bonn, Germany; 6 Department of Clinical Epidemiology and Applied Biostatistics, University Hospital Tübingen, Tübingen, Germany; 7 Department of Medical Biostatistics, Epidemiology and Informatics, University of Mainz, Mainz, Germany; 8 Department of Radiotherapy and Radiation Oncology, University Hospital Mainz, Mainz, Germany; 9 Department of Urology, Klinikum Leverkusen, Leverkusen, Germany; 10 Center for Urology, Lübeck, Germany; 11 Department of Urology, Klinikum Lippe Detmold, Detmold, Germany; 12 Department of Urology, Vinzenzkrankenhaus, Hannover, Germany; 13 Department of Urology, University Hospital Homburg/Saar, Homburg, Germany

**Keywords:** Prostate cancer, Randomized clinical trial, Prostatectomy, Active surveillance, External beam radiotherapy, Permanent seed implantation

## Abstract

**Purpose:**

The optimal treatment for patients with low to early-intermediate risk prostate cancer (PCa) remains to be defined. The randomized PREFERE trial (DRKS00004405) aimed to assess noninferiority of active surveillance (AS), external-beam radiotherapy (EBRT), or brachytherapy by permanent seed implantation (PSI) vs. radical prostatectomy (RP) for these patients.

**Methods:**

PREFERE was planned to enroll 7600 patients. The primary endpoint was disease specific survival. Patients with PCa stage ≤ cT2a, cN0/X, M0, PSA ≤ 10 ng/ml and Gleason-Score ≤ 3 + 4 at reference pathology were eligible. Patients were allowed to exclude one or two of the four modalities, which yielded eleven combinations for randomization. Sixty-nine German study centers were engaged in PREFERE.

**Results:**

Of 2251 patients prescreened between 2012 and 2016, 459 agreed to participate in PREFERE. Due to this poor accrual, the trial was stopped. In 345 patients reference pathology confirmed inclusion criteria. Sixty-nine men were assigned to RP, 53 to EBRT, 93 to PSI, and 130 to AS. Forty patients changed treatment shortly after randomization, 21 to AS. Forty-eight AS patients with follow-up received radical treatment. Median follow-up was 19 months. Five patients died, none due to PCa; 8 had biochemical progression after radical therapy. Treatment-related acute grade 3 toxicity was reported in 3 RP patients and 2 PSI patients.

**Conclusions:**

In this prematurely closed trial, we observed an unexpected high rate of termination of AS and an increased toxicity related to PSI. Patients hesitated to be randomized in a multi-arm trial. The optimal treatment of low and early-intermediate risk PCa remains unclear.

**Electronic supplementary material:**

The online version of this article (10.1007/s00432-020-03327-2) contains supplementary material, which is available to authorized users.

## Introduction

The incidence of newly detected prostate cancer (PCa) in Germany was 63.710 in 2012 (Robert-Koch-Institute 2016). Three quarters of these cases were cT1 and cT2 tumors. The optimal therapy for low to (early) intermediate risk-profile PCa is still under debate (Mottet et al. 2017; Sanda et al. 2018).

One randomized clinical trial (RCT), SPCG-4 compared watchful-waiting (WW) with radical prostatectomy (RP) in 695 T1-T2 (early) PCa patients. After a median of 23.6 years follow-up, overall survival (OS), disease specific survival (DSS), and freedom from metastasis were significantly better in the active treatment arm than with WW. However, after 18 years, one third of the WW patients did still not require androgen deprivation therapy (ADT) (Bill-Axelson et al. 2005; Bill-Axelson et al. 2014; Bill-Axelson et al. 2018).

In the PIVOT trial, with 731 patients (43% low risk, 36% intermediate risk, 20% high-risk), and median 12.7 years follow-up, no significant improvement of DSS or OS was reported for RP vs WW. However, a high comorbidity and associated mortality impair the validity of these results (Wilt et al. 2009, 2012, 2017).

In the ProtecT study, 1.643 cT1-cT2 PCa patients were randomized between active monitoring, RP, and external beam radiotherapy (EBRT) with short-term ADT. After 10 years, OS and DSS did not differ between the three arms (all > 98.8%), but progression-free rates was improved after active treatment (Donovan et al. 2016; Hamdy et al. 2016). Brachytherapy, which is also recommended for low risk and selected intermediate-risk PCa by the German and European guidelines (Mottet, Bellmunt et al. 2017, Leitlinienprogramm Onkologie der Arbeitsgemeinschaft der Wissenschaftlichen Medizinischen Fachgesellschaften e.V. (AWMF) 2018) was not investigated in the ProtecT trial.

The German PREFERE trial (DRKS00004405) was the first RCT that aimed to show non-inferiority vs. RP in disease-specific survival of all other guideline-recommended first-line treatment approaches for PCa with low to early-intermediate risk (Mottet, Bellmunt et al. 2017, Leitlinienprogramm Onkologie der Arbeitsgemeinschaft der Wissenschaftlichen Medizinischen Fachgesellschaften e.V. (AWMF) 2018), namely EBRT, brachytherapy by permanent seed implantation (PSI), and active surveillance (AS) using DSS as primary endpoint. Moreover, PREFERE aimed to assess the patients’ treatment preferences by offering randomization between two, three or all four trials arms. In addition to these aspects, we report on adherence specifically to AS, on treatment related toxicity and on quality of life.

## Patients and methods

PREFERE (Sponsor ID: 2011-PCA-01) was initiated and financed by institutions involved in the German health care system, including the German Cancer Aid, the National Association of Health Insurances, and the German Cancer Society. Sixty-nine certified PCa centers participated in the trial. Each center had to fulfill predefined quality criteria including a minimum case number for all four treatment concepts. The site of the primary investigator started recruiting in September 2012.

**Fig. 1:**
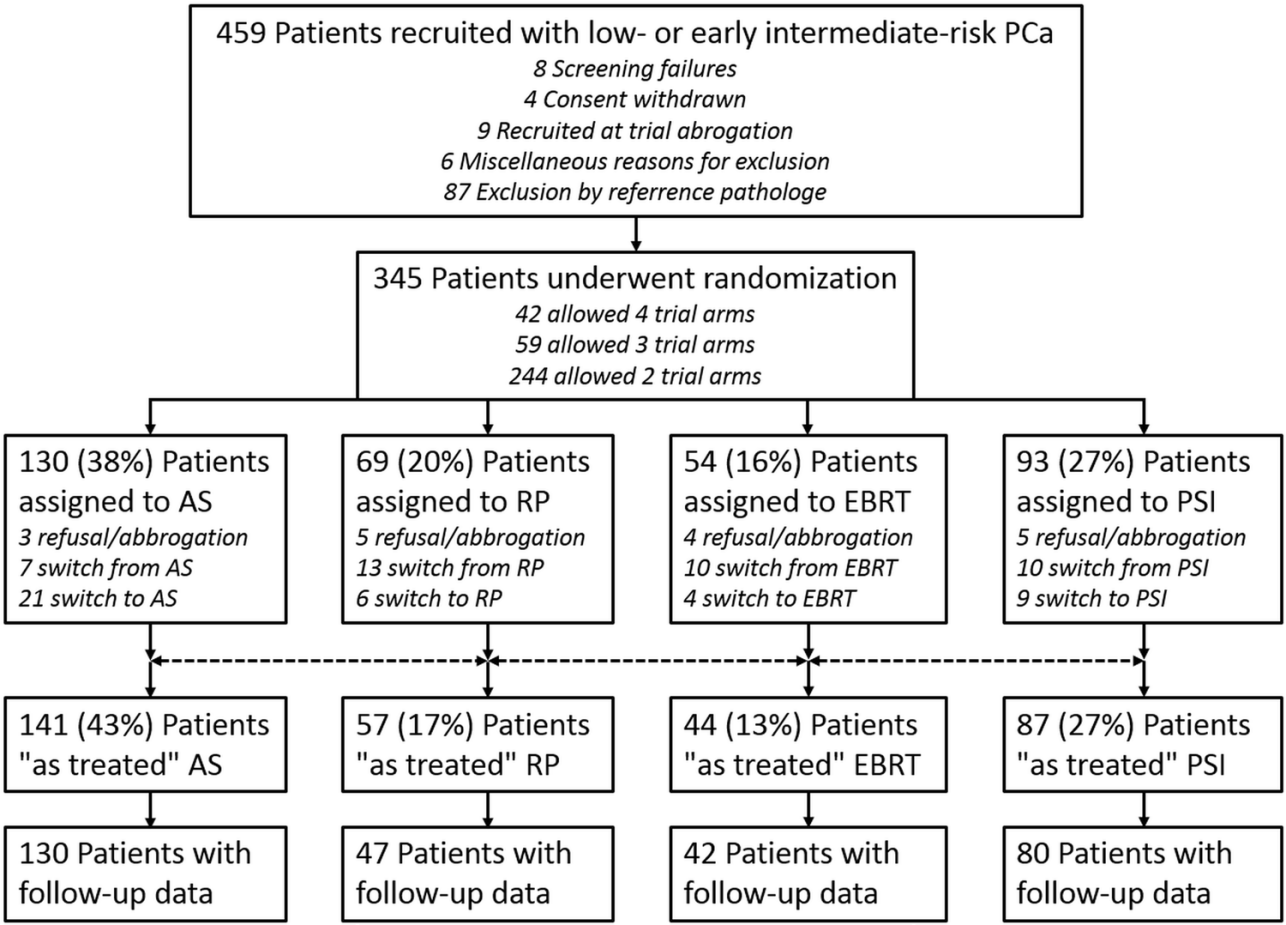
Flow chart of the PREFERE trial, recruiting from September 2012 to December 2016. *AS* active surveillance, *RP* radical prostatectomy, *EBRT* External beam radiotherapy, *PSI* Permanent seed implantation

Men aged 18–75 years were eligible for PREFERE, if they had ≤ cT2a PCa (comprising ≤ 30% positive of all biopsy scores and ≤ 5 mm of continuous tumor tissues), cN0/X, M0, a PSA ≤ 10 ng/ml, Gleason-Score (GS) ≤ 7a (3 + 4), ECOG performance status 0 or 1, and IPSS-Score < 18. Per protocol, treatment had to start within 6 months after pathological PCa confirmation. All primary biopsies had to be submitted to reference pathology to obtain a second expert’s opinion, prior to randomization (Kristiansen et al. 2013). The randomization scheme/flow chart is shown in Fig. 1.

RP could be conducted as open or laparoscopic (including robot-assisted) surgery, with limited pelvic lymphadenectomy for GS 7a, to estimate the proportion of positive lymph nodes in early-intermediate risk patients. EBRT was given as image guided intensity modulated radiotherapy (IGRT/IMRT). For low-risk patients the prescribed dose was 76 Gy to the prostate. For early-intermediate risk patients, the dose was 78 Gy including 58 Gy to the base of the seminal vesicles. PSI was carried out under intraoperative real-time control and planning. The prescribed dose was 145 Gy using J-125 seeds. AS included a confirmatory biopsy at 6 months, re-biopsy after 12 months for GS 6, after 3 and 12 months for GS 7a, and every three years later on. For all trial arms, the scheduled follow-up including PSA sampling was as recommended in EAU guidelines (Mottet et al. 2017). AS was terminated only in cases of tumor grade and/or volume progression or if the patient did not wish to continue. The planned minimum follow-up time was 13 (max 17) years. Side effects were documented based on CTCAE (version 4.0) criteria.

PREFERE was in fact an RCT stratified for 11 different preference patterns. In each stratum, patients were randomly allocated to one out of two, three, or all four treatment options. Each patient choose “his” specific subtrial according to personal preferences. The primary endpoint of PREFERE was DSS. One objective was to establish noninferiority of AS, EBRT and PSI to RP in terms of DSS. For each of these three comparisons, the noninferiority margin for 13 years survival was set to 5%. Sample size planning (*N* = 7.600) was subject to the requirement that the one-sided log-rank test for noninferiority (Wellek 2010) at a multiplicity-corrected significance level of 1.67% should provide a power of 90% against the alternative that the probability of not dying from PCa within 13 years is 90%.

Secondary endpoints included OS, biochemical progression (post-RP: persisting or rising above 0.2 ng/ml, post-RT: 2 ng/ml above the nadir), distant metastases, treatment-related toxicity and quality of life. Progression under AS had to be confirmed by re-biopsy. Quality of life was assessed with the European Organisation for Research and Treatment of Cancer (EORTC) 30-item Quality of Life Core Questionnaire (QLQ-C30) (Aaronson et al. 1993). The QLQ-C30 items were transformed into a global health scale and five functioning scales (emotional, physical, cognitive, social, and role), as well as three multi-item and six single-item symptom scales. Further, patients were asked to fill QLQ-PR25 which includes questions on sexual activity and function (van Andel et al. 2008; van Leeuwen et al. 2017). QLQ reports were scheduled within 3 months post-randomization, and then after 1, 2, 3, 5, 7, 10, 13, and 17 years.

A professional 1-hour video explaining the character of early-stage prostate cancer, the treatment procedures, and the aims of PREFERE was offered to all patients (Sanger et al. 2015). A systematic external monitoring of the treatment was performed and a quality assurance (QA) program for all study data was predefined by the trial protocol. The ethics committee of the Medical Council of Saarland gave central approval (184/12) for the trial. Additionally, local ethics approval was obtained by participating centers.

## Results

PREFERE was prematurely closed due to poor recruitment. Of 2251 prescreened patients, 459 were submitted to reference pathology and in 345 inclusion criteria were confirmed and they were randomized (patient characteristics shown in Table 1). Of these patients, 42 (12%) accepted all four treatment options, 59 (17%) excluded one and 244 (71%) excluded two arms. After randomization, 12% of the patients decided to change from their assigned treatment. Immediate change within one months occurred in 5% of men randomized to AS, in 19% of RP, 19% of EBRT, and 11% of PSI patients, respectively (Fig. 1).

**Table 1:** PREFERE patients’ characteristics

Item	Characteristic	*N*	%
Age (*n* = 345)	< 65 years	160	46
	65—70 years	89	26
	71—75 years	96	28
Initial PSA (*n* = 345)	≤ 6 ng/ml	181	52
	> 6 ng/ml	164	48
Gleason Score (*n* = 345)	≤ 6	225	65
	7a	120	35
Accepted treatment strategy (*n* = 345)	RP	168	49
	EBRT	152	44
	PSI	225	65
	AS	289	84
Health insurance (*n* = 340)	Public	291	86
	Private	49	14
Erectile dysfunction at recruitment (*n* = 333)	Grad 0	217	65
	Grad 1	67	20
	Grad 2	30	9
	Grad 3	19	6
Urinary incontinence at recruitment (*n* = 333)	Grad 0	327	98
	Grad 1	6	2
Rectal problems/disorders at recruitment (*n* = 341)	Grad 0	336	99
	Grad 1	5	1

AS: active surveillance; EBRT: external beam radiotherapy; PSI: permanent seed implantation; RP: radical prostatectomy

**Fig. 2:**
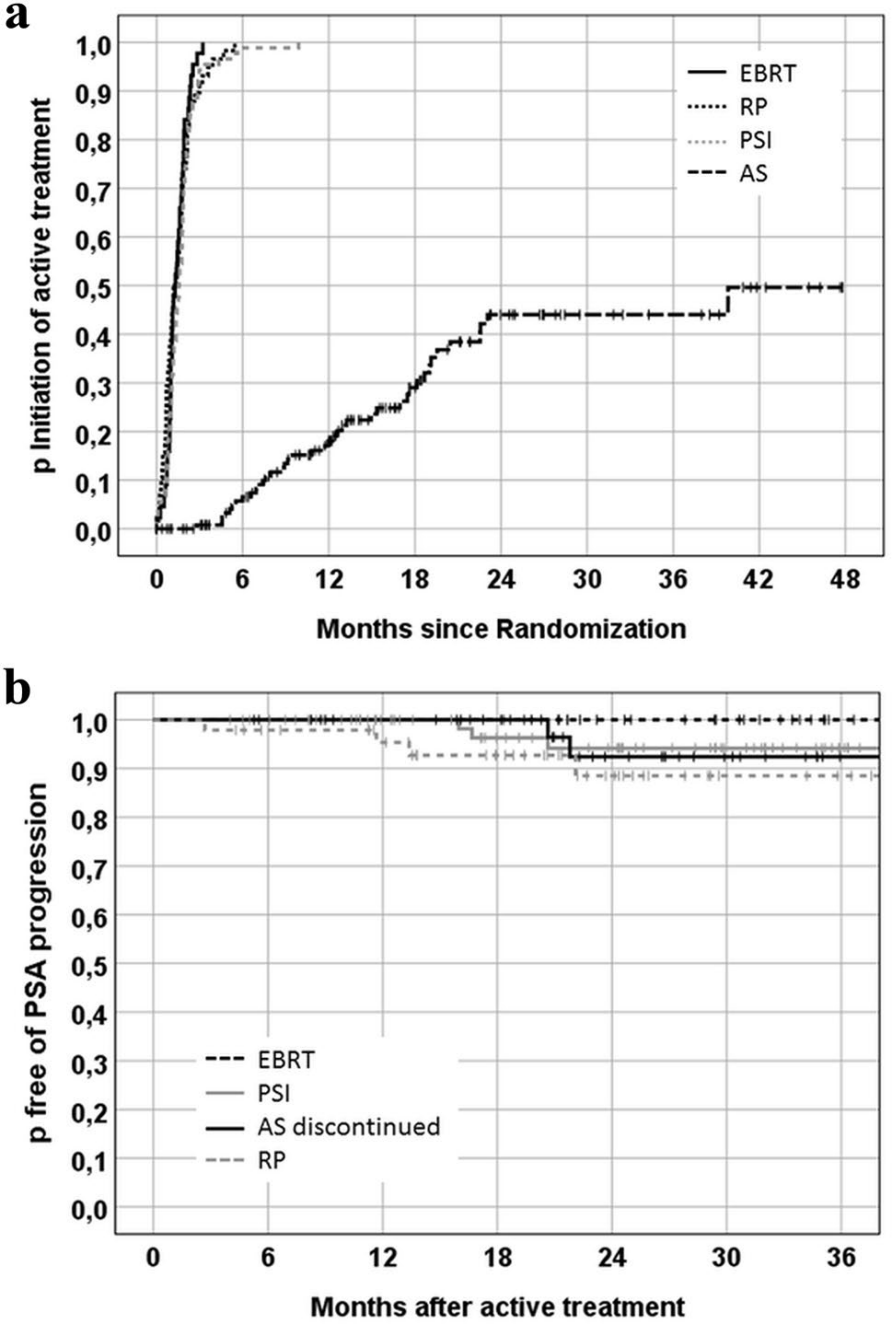
Kaplan–Meier estimates for **a** the cumulative probability of undergoing radical intervention during the follow-up period, stratified by treatment, including immediate changes. AS curve: Active treatment for confirmed progression; **b** freedom from biochemical progression. For patients who discontinued active surveillance, analysis starts at the time of active treatment. AS: active surveillance; EBRT: external beam radiotherapy; PSI: permanent seed implantation; RP: radical prostatectomy

The median follow-up was 19.7 (max. 59.8) months. During the reported period, five patients died, none of PCa. Biochemical progression was documented for 8 patients after immediate active treatment. Of 141 “as treated” AS patients, 56 experienced biopsy confirmed progression and 48 received active treatment (2-years rate for GS 6: 35%, GS 7a: 66%, overall: 44%; Fig. 2a) after which 2 men had further progression. Figure 2b shows the Kaplan-Maier probabilities for biochemical progression after radical intervention stratified by treatment arm.

Despite the efforts to advertise the trial as an opportunity for patients to benefit from the high standards of an RCT, PREFERE had trial-limiting recruiting problems. Of the 459 patients who were willing to participate, 114 had to be excluded, 87 (19% of all patients) due to findings from reference pathology. Of the latter, 23% were excluded because the primary report did not allow verification of histological inclusion criteria (e.g. tumor extent reported in percent, whereas the study required absolute measures in mm). In another 21% of exclusions, the local sites misinterpreted a pathology report and referred cases with obvious exclusion criteria (e.g. Gleason Score 4 + 3 = 7b) to reference pathology. The remaining exclusions (11% in total) resulted from differences between local and central pathology. A false-positive carcinoma diagnosis was seen in 7 cases (1.5%). As expected, a considerable inter-observer variability was seen in Gleason scoring, and especially in the discrimination of ISUP Grade Groups 1 vs 2.

**Fig. 3:**
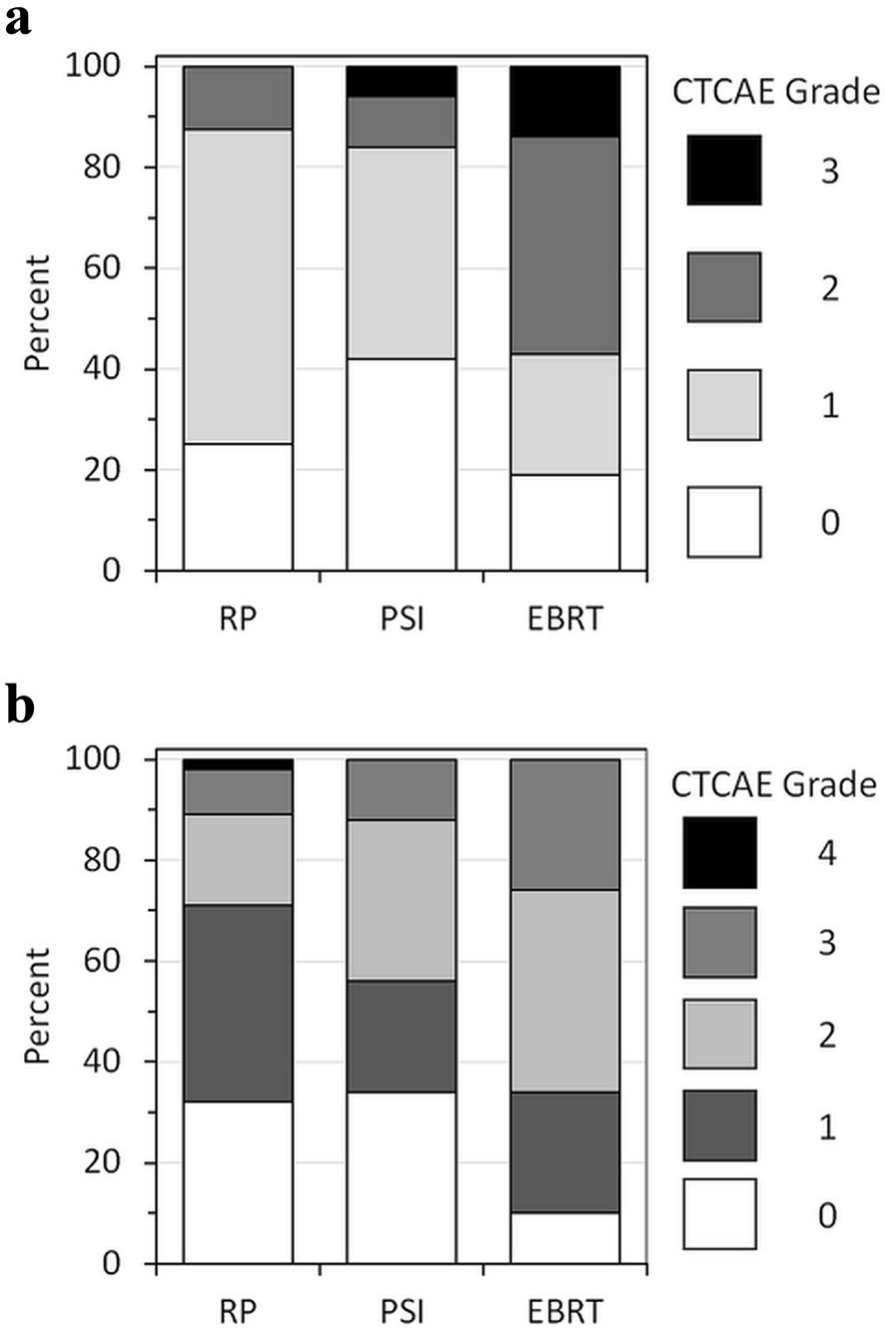
Toxicity (CTCAE) after radical treatment, including patients who discontinued active surveillance. **a** Acute toxicity (*N* = 220); **b** toxicity 12 months after radical treatment (*N* = 178). RP: radical prostatectomy; PSI: permanent seed implantation; EBRT: external beam radiotherapy

Figure 3a shows the distribution of acute toxicity after radical intervention in 220 patients (including men who discontinued from AS). CTCAE grade ≥ 2 events were reported for 26 patients. Grade 3 events occurred in 2 patients after PSI and in 3 patients after RP. At 12 months follow-up (*N* = 178), 55 patients experienced late grade 2 reactions. Grade ≥ 3 toxicity occurred in 15 RP, 9 PSI and 4 EBRT patients, respectively (Fig. 3b).

**Fig. 4:**
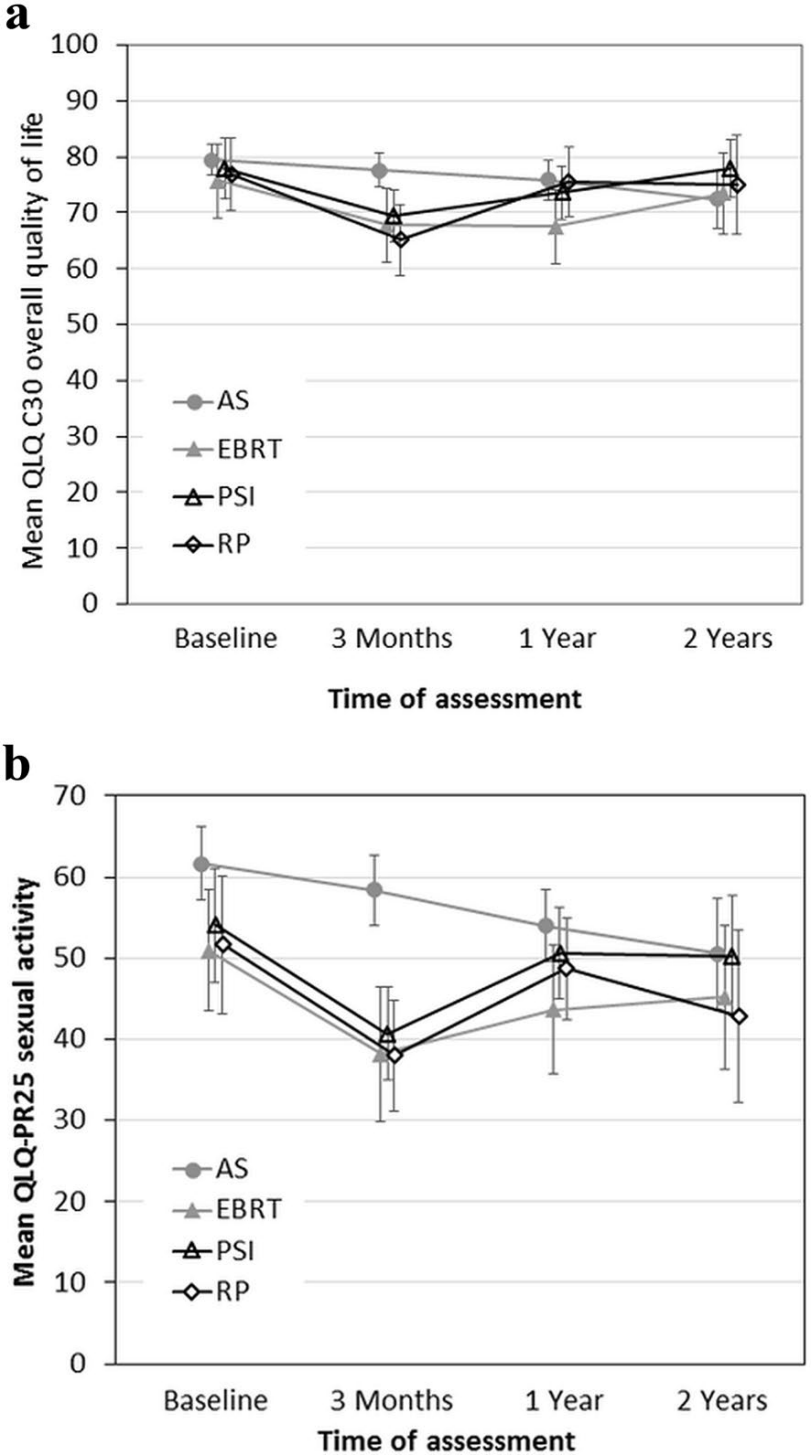
Quality of life of PREFERE patients at various times after trial entry (including men who changed trial arm immediately after randomization). **a** Overall quality of life according to QLQ C30 questionnaires; **b** sexual activity according to QLQ PR 25 questionnaires. Mean values with 95% confidence intervals are shown. Identical *X*-values slightly offset to improve legibility. *AS* active surveillance, *EBRT* external beam radiotherapy, *PSI* permanent seed implantation, *RP* radical prostatectomy

Quality of life was evaluated from the 1042 questionnaires of 180–321 patients who reported at various times of the trial. Figure 4a, b show the average global health score and sexual activity, respectively. Decreasing scores in the AS arm may partly relate to changes to active treatment.

Study review revealed more quality-assurance issues in PSI than in RP and EBRT. This included dose errors (so far, none leading to increased toxicity or recurrence) and missing/incomplete post-planning documentation (19%). In AS, 25% of the scheduled follow-up dates and 25–50% of re-biopsies beyond the first one were left out.

## Discussion

PREFERE was the first phase III trial to investigate all four guideline-recommended treatment options (RP, EBRT, PSI, AS) for patients with low- to early-intermediate risk PCa. Due to the premature termination of the PREFERE trial, the results on oncological endpoints can only be fragmentary. The follow-up of the recruited 345 patients could be extended to February 2018.

In spite of the limited number of PREFERE patients, some observations are remarkable: The rate of men who switched from AS to radical treatment (44% at 2 years) was twice as high as in the ProtecT trial (Hamdy et al. 2016). A survey based on the Movember Foundation’s global GAP3 database reported on 10,296 men on AS from 21 centers across 12 countries. At two years, 15% of the patients discontinued AS due to progression, 6% opted for active treatment without evidence of progression, and 1% changed to watchful waiting. It took nearly 5 years until 40% had switched from AS (Van Hemelrijck et al. 2019), a proportion observed in PREFERE after less than 2 years. The high number of follow-up visits including three confirmation biopsies in PREFERE within the first 24 months may have contributed to this high discontinuation rate.

So far, the progression-free rates seem to be comparable to published data (Kittel et al. 2015; Hamdy et al. 2016; Gestaut et al. 2017). However, given the short follow-up, the assessment even of this endpoint with 10 events can only be tentative.

The considerable rate of exclusions based on reference pathology underlines the role of central pathological review regarding standardized histological inclusion criteria for prospective trials. Even though the rate of serious errors (e.g. false-positive carcinoma diagnosis) matches published data (Chun et al. 2008; Wolters et al. 2010), higher rates of interobserver variability became dominantly apparent for ISUP gradings. It is important news for patients eligible for AS, that a second opinion pathology by an expert reference center may substantially improve outcomes in AS. Specifically, the definition of Gleason pattern 4 (and its quantification) appears to be problematic, leading to diagnostic fuzziness in the distinction of ISUP Grade Groups 1–3 [GS ≤ 7a (included) vs GS ≥ 7b (excluded)] represented a major problem. Dedicated training courses may help to reduce discrepancies between local and reference pathology.

Overall, acute toxicity after treatment was acceptable with < 3% grade ≥ 3 events. However, post- RP, the incidence was 6% (5/83), which is comparable to published data (Hugosson et al. 2011). Interestingly, the preliminary data suggest an increased risk of toxicity after PSI compared with EBRT. With quality assurance problems including inhomogeneous and even insufficient doses, oncological outcomes after PSI are unlikely to become superior to EBRT at the current state.

QLQ assessments showed an early decline of global health and sexual activity after definitive treatment which is in line with a systematic review on quality-of-life outcomes after primary treatment for clinically localized PCa (Lardas et al. 2017). However, with the steadily growing number of AS patients switching to definitive treatment with corresponding sequelae, QLQ values decline after AS, too, and the data of all four arms converged after 1 year.

The problems that led to the early termination of PREFERE deserve a critical consideration. According to a systematic analysis of entries on phase II–III studies in the registry ClinicalTrial.gov, poor accrual is the most frequent single cause of early termination of trials (38.7%), contributing more than logistic problems including cancelation by sponsors (20.5%), toxicity (18.1%), or other reasons like becoming obsolete (22.7%) (Stensland et al. 2014). The recruiting problems in PREFERE had several reasons: Patients probably hesitated to be randomized in a situation where they rather expected recommendations by their physicians, hoping for the optimal treatment (which to determine would have been the goal of this RCT). Others had undisputable treatment preferences (frequently favoring RP), based on publicly available information on PCa, or on suggestions from their family/social environment. An additional obstacle may have been a campaign launched in part in lay media, which declared AS the standard of care and denied the ethic justification of a trial that offered primary active treatment.

In fact, the meanwhile published ProtecT data raise doubts about the equiefficacy of AS versus primary active treatment to prevent progression to an incurable tumor stage (Hamdy et al. 2016). However, this trial’s sample size was originally calculated to show superiority of RP vs EBRT + ADT and AS (Lane et al. 2014). Its results may therefore have to be interpreted with caution. PSI was not an option in ProtecT. As we thus do not have clear evidence towards the optimal therapy for low to intermediate-risk PCa, treatment alternatives require careful consideration in future studies.

The problems with quality assurance and the observed toxicity of PSI must be addressed. Complications after EBRT may improve with image guidance and arc techniques (VMAT, RapidArc). RP patients may benefit from robot-assisted surgery. Published RCTs comparing AS and immediate treatment do not give unequivocal results. Specifically, early-intermediate risk patients were usually not included. In addition, PSI has so far not been investigated in such trials.

The proportion of 43% of patients in the AS arm of PREFERE underlines the importance of optimizing compliance with this strategy including its tight follow-up schedule (Kinsella et al. 2018). The 10% rate of migration between treatment arms and the complex pattern of the changes may have implications for future multiple-arm studies. If migration occurs massively in favor of one arm, statistical requirements may be violated.

Messages from PREFERE

- While most patients preferred randomization in groups including AS, an unexpectedly high rate switched from AS to radical treatment within 2 years.
- AS initially delayed a reduction in quality of life, but QLQ scores for all trial arms converged after 1 year, partly due to changes from AS to active treatment.
- Exclusions from trial based on reference pathology suggests this mode of quality control to be essential for RCTs relying on histopathological criteria.

## Electronic supplementary material

Below is the link to the electronic supplementary material.

Supplementary file1 (PDF 273 kb)
