# Supplementary material for: Results of a randomized trial of treatment modalities in patients with low or early-intermediate risk prostate cancer (PREFERE trial)
Source: J Cancer Res Clin Oncol. 2020 Sep 4;147(1):235–42. doi: 10.1007/s00432-020-03327-2 (PMC7810635; doi:10.1007/s00432-020-03327-2)
Supplement: Supplementary file 1 — Supplementary file1 (PDF 273 kb) [file 432_2020_3327_MOESM1_ESM.pdf]

Results of a Randomized Trial of Treatment Modalities in Patients with Low or Early-Intermediate Risk Prostate Cancer (PREFERE trial)

Thomas Wiegel [a*], Peter Albers [b], Detlef Bartkowiak [a], Roswitha Bussar-Maatz [c], Martin Härter [d], Glen Kristiansen [e], Peter Martus [f], Stefan Wellek [g], Heinz Schmidberger [h], Klaus Grozinger [i], Peter Renner [j], Fried Schneider [k], Martin Burmester [l], Michael Stöckle [m]

[a] *Department of Radiotherapy and Radiation Oncology, University Hospital Ulm, Ulm, Germany*
[b] *Department of Urology, University Hospital Düsseldorf, Düsseldorf, Germany*
[c] *PREFERE Project Management, German Cancer Society, Berlin, Germany*
[d] *Department of Medical Psychology, University Medical Center Hamburg-Eppendorf, Hamburg, Germany*
[e] *Institute of Pathology, University Hospital Bonn, Bonn, Germany*
[f] *Department of Clinical Epidemiology and applied Biostatistics, University Hospital Tübingen, Tübingen, Germany*
[g] *Department of Medical Biostatistics, Epidemiology and Informatics, University of Mainz, Mainz, Germany*
[h] *Department of Radiotherapy and Radiation Oncology, University Hospital Mainz, Mainz, Germany*
[i] *Department of Urology, Klinikum Leverkusen, Leverkusen, Germany*
[j] *Center for Urology, Lübeck, Germany*
[k] *Department of Urology, Klinikum Lippe Detmold, Detmold, Germany*
[l] *Department of Urology, Vinzenzkrankenhaus, Hannover, Germany*
[m] *Department of Urology, University Hospital Homburg/Saar; Germany*

*Corresponding author: Thomas Wiegel, MD
E-Mail: thomas.wiegel@uniklinik-ulm.de

Supplement 1: Members of the PREFERE study group in alphabetical order.

Peter Albers, Universitätsklinik Düsseldorf; Andreas Auge, SRH Wald Klinikum Gera; Detlef Bartkowiak, Universitätsklinikum Ulm; Eduard Becht , Krankenhaus Nordwest, Frankfurt am Main; Hanjo Belz, Zeisigwaldkliniken Bethanien Chemnitz; Knut Bescherer, Carl Thiem Klinikum Cottbus; Christian Bolenz, Universitätsklinikum Ulm; Stephanie Böttcher, Helios Klinikum Wuppertal; Dirk Bottke, Universitätsklinikum Ulm; Björn Boysen, Universitätsklinik Rostock; Moritz Braun, Heilig Geist Krankenhaus, Köln; Martin Burmester, Vinzenzkrankenhaus Hannover; Stephan Buse, Alfried Krupp Krankenhaus, Essen; Wolfgang Diederichs, BG Klinikum Unfallkrankenhaus Berlin; Christoph Durek, Urologisches Zentrum Lübeck; Felix Engelhardt, Klinikum der Stadt Ludwigshafen; Dirk Fahlenkamp, Zeisigwaldkliniken Bethanien Chemnitz; Priska Fettweis, Universitätsklinikum Carl Gustav Carus, Dresden; Jan Fichtner, Evangelisches und Johanniter Klinikum, Oberhausen; Marcel Fiedler, SLK Kliniken Heilbronn Klinikum am Gesundbrunnen; Arnt René Fischedick, Clemenshospital, Münster; Tobias Fritsch, HELIOS Klinikum Erfurt; Detlef Frohneberg, Städtisches Klinikum Karlsruhe; Rolf Gillitzer, Klinikum Darmstadt; Markus Graefen, Universitätsklinikum Hamburg Eppendorf; Klaus Grozinger, Klinikum Leverkusen; Nicole Grunert, Bundeswehrkrankenhaus Ulm; Alfons Gunnemann, Klinikum Lippe Detmold; Ulrich Haag, Klinikverbund Südwest Kliniken Nagold, Akademisches Lehrkrankenhaus; Carolin Eva Hach, Alfried Krupp Krankenhaus, Essen; Axel Häcker, Universitätsklinikum Mannheim; Oliver Hakenberg, Universitätsklinik Rostock; Peter Hammerer, Städtisches Klinikum Braunschweig; Gencay Hatiboglu, Universitätsklinikum Heidelberg; Siegfried Heida, Klinikum Nordoberpfalz, Standort Klinikum Weiden; Torsten Heil, SRH Wald

Klinikum Gera; Thomas Heinrich, HELIOS Klinikum Erfurt; Jan Herden, Universitätsklinikum Köln; Jannis Hildebrandt, Dr. Horst Schmidt Kliniken, Wiesbaden; Reinhard Hofmann, Klinikum Wolfsburg; Markus Hohenfellner, Universitätsklinikum Heidelberg; Bernd Hoschke, Carl Thiem Klinikum Cottbus; Ulrich Humke, Klinikum Stuttgart Katharinenhospital; Thomas Jachertz, Städtisches Klinikum Lüneburg; Alexandra John, Städtisches Klinikum Karlsruhe; Tilman Kälble, Klinikum Fulda; Jürgen Keil, Krankenhaus der Barmherzigen Brüder, Trier; Rüdiger Klän, Klinikum Gütersloh; Klaus Kleinschmidt, Dr. Horst Schmidt Kliniken, Wiesbaden; Theodor Klotz, Klinikum Nordoberpfalz, Standort Klinikum Weiden; Jennifer Kranz, St. Antonius Hospital Eschweiler; Waldemar Krings, Kliniken Maria Hilf, Mönchengladbach; Holger Kujau, SRH Wald Klinikum Gera; Jens Küster, Klinikum Hann. Münden; Roland Lang, Urologische Klinik München; Jan Lehmann, Städtisches Krankenhaus Kiel; Armin Leitenberger, Klinikum Wolfsburg; Stefan Machtens, Marien Krankenhaus, Bergisch Gladbach; Lukas Manka, Städtisches Klinikum Braunschweig; Dimitrios Manos, Vivantes Klinikum Am Urban, Berlin; Stefan Marschner, Heilig Geist Krankenhaus, Köln; Michaela Metz, Universitätsklinikum Würzburg; Michael Metze, Klinikum Salzgitter; Maurice Stefan Michel, Universitätsklinikum Mannheim; Kurt Miller, Charité Campus Benjamin Franklin, Berlin; Arndt Christian Müller, Universitätsklinik Tübingen; Yamini Nagaraj, Universitätsklinikum Hamburg Eppendorf; Michael Noe, Missionsärztliche Klinik, Würzburg; Ralph Oberneder, Urologische Klinik München; Carsten Henning Ohlmann, Universitätsklinik des Saarlandes, Homburg; Roberto Olianas, Städtisches Klinikum Lüneburg; Stefan Orth, Klinikum Westfalen (Knappschaftskrankenhaus), Dortmund; Alexander Ottenhof, Johannes Wesling Klinikum Minden; Maik Pechoel, Klinikum der Ernst Moritz Arndt Universität Greifswald; Hansjürgen Piechota, Johannes Wesling Klinikum Minden; Bülent Polat, Universitätsklinikum Würzburg; Chris Protzel, Universitätsklinik Rostock; Robert Rabenalt, Universitätsklinik Düsseldorf; Jens Rassweiler, SLK Kliniken Heilbronn Klinikum am Gesundbrunnen; Frank Reiher, AMEOS Klinikum Haldensleben; Peter Renner, Urologisches Zentrum Lübeck; Roger Riexinger, Klinikverbund Südwest Kliniken Nagold, Akademisches Lehrkrankenhaus; Jan Roigas, Vivantes Klinikum Am Urban, Berlin; Stephan Roth, Helios Klinikum Wuppertal; Arne Schlattmann, Marien Krankenhaus, Bergisch Gladbach; Fried Schneider, Klinikum Lippe Detmold; Christian Schoop, Klinikum Gütersloh; Martin Schostak, Universitätsklinik Magdeburg; Melanie Schremmer, Klinikum Fulda; Anne Schröder, AMEOS Klinikum Haldensleben; Wolfgang Schultze Seemann, Universitätsklinikum Freiburg; Miguel Garcia Schürmann, Marienhospital Wesel; Axel Semjonow, Universitätsklinikum Münster; Jörg Sommer, St. Franziskus Hospital Lohne; Hans Jörg Sommerfeld, Marien Hospital, Marl; Christoph Sparwasser, Bundeswehrkrankenhaus Ulm; Andreas Stammel, Marienhospital Wesel; Joachim Steffens, St. Antonius Hospital Eschweiler; Ursula Steiner, Charité Campus Benjamin Franklin, Berlin; Thomas Steiner, HELIOS Klinikum Erfurt; Michael Stöckle, Universitätsklinik des Saarlandes, Homburg; A. Strauß, Universitätsmedizin Göttingen Georg August Universität; Petra Strölin, Universitätsklinikum Hamburg Eppendorf; Caroline Wedel, Imland Klinik Rendsburg; Peter Weib, Jung Stilling Krankenhaus Siegen; Klaus Weichert Jacobsen, Klinikum Herford; Karl Weingärtner, Klinikum am Bruderwald, Bamberg; Evelyn Weinstrauch, Johanniter Krankenhaus Genthin Stendal; Johannes Weißmüller, Klinikum Aschaffenburg; Thomas Wiegel, Universitätsklinikum Ulm; Felix Wiesend, Klinikum Stuttgart Katharinenhospital; Joerg Winkle, Klinikum Aschaffenburg; Alexander Winter, Klinikum Oldenburg; Manfred P. Wirth, Universitätsklinikum Carl Gustav Carus, Dresden; Ulrich Witzsch, Krankenhaus Nordwest, Frankfurt am Main; Wigand Wucherpfennig, Klinikum Salzgitter; Stefan Zastrow, Universitätsklinikum Carl Gustav Carus, Dresden; Jürgen Zumbé, Klinikum Leverkusen
